# Pharmacokinetics, Safety, and Tolerability of (R)-Ketamine Hydrochloride Injection, a Novel Rapid-Acting Antidepressant, in Healthy Chinese Subjects

**DOI:** 10.3390/ph18071079

**Published:** 2025-07-21

**Authors:** Rui Wang, Yuqian Yang, Tong Zhou, Bingjie Zou, Li Ding

**Affiliations:** 1 Department of Pharmaceutical Analysis, China Pharmaceutical University, Nanjing 211198, China; rain.927@163.com (R.W.); 3323010546@stu.cpu.edu.cn (Y.Y.); 2 Jiangsu Nhwa Pharmaceutical Co., Ltd., Xuzhou 221400, China; sandratong@foxmail.com; 3 Nanjing Clinical Technology Co., Ltd., Nanjing 211198, China

**Keywords:** (R)-ketamine, (R)-norketamine, treatment-resistant depression, N-methyl-D-aspartate receptor antagonist, pharmacokinetics, safety

## Abstract

**Objectives**: (R)-ketamine hydrochloride injection is a novel, rapid-acting antidepressant for the treatment of treatment-resistant depression. The aim of this study was to assess the pharmacokinetics, safety, and tolerability of (R)-ketamine hydrochloride injection in healthy Chinese subjects following ascending single intravenous doses ranging from 10.0 mg to 180 mg. **Methods**: This randomized, double-blind, placebo-controlled study was conducted in 50 healthy male and female Chinese subjects after single ascending doses of (R)-ketamine hydrochloride injection (10.0, 30.0, 60.0, 120, and 180 mg). Ten subjects (including two subjects treated with a placebo) were included in each dose cohort. Pharmacokinetic characteristics, safety, and tolerability profiles of the study drug were evaluated. **Results:** After the intravenous doses administered from 10.0 mg to 180 mg of (R)-ketamine hydrochloride injection to the subjects, the C_max_ and AUC values for both (R)-ketamine and its metabolite (R)-norketamine in the subjects increased approximately proportionally to the doses. The average peak plasma concentration levels at the five dose cohorts ranged from 56.0 to 1424 ng/mL and 27.7 to 491 ng/mL for (R)-ketamine and (R)-norketamine, respectively. The adverse events of (R)-ketamine hydrochloride injection were temporary and recovered spontaneously without treatment. **Conclusions**: In summary, (R)-ketamine hydrochloride injection was safe and well tolerated in healthy Chinese subjects. The clinical study results laid a foundation for the further clinical studies of (R)-ketamine hydrochloride injection in patients.

## 1. Introduction

Major depressive disorder (MDD) is a neuropsychiatric condition characterized by persistent and recurrent episodes of low mood, pain, and despair, as well as feelings of pessimism and, in severe cases, suicidal ideation [1]. A large portion of patients with MDD (ca. 30%) are considered to have treatment-resistant depression (TRD) following inadequate responses after two or more successive treatment trials of first-line antidepressants [2]. TRD is a severe, potentially life-threatening condition associated with a high risk of suicide, with over 20% of patients having made at least one suicide attempt during their lifetime [3]. Patients with TRD often require treatment with multiple antidepressant medications, which can elevate the risk of drug side effects and increase the costs of hospitalization and medical care, ultimately leading to a decline in the patients’ quality of life [4]. Therefore, relieving the symptoms of TRD patients and reducing the risk of suicide are particularly important in clinical treatment.

Over the past decade, two new developments in the medical treatment of TRD have been identified: typical serotonergic (e.g., *psilocybin*, ayahuasca) and atypical glutamatergic psychedelics (e.g., ketamine, (S)-ketamine). Most of researches focus on ketamine and (S)-ketamine [5]. In 2000, researchers serendipitously discovered that intravenous ketamine could alleviate symptoms of patients with MDD and quickly reduce their suicidal ideation [6], which marked the most significant breakthrough in the field of antidepressants over the past six decades. In 2006, a randomized, placebo-controlled, double-blind cross-over study replicated this breakthrough finding. The researchers confirmed that a single injection of ketamine (0.5 mg/kg, over 40 min) could rapidly exert antidepressant effects within 2 h, and its efficacy could last for at least 1 week in TRD patients [7]. In addition, electroconvulsive therapy (ECT) has long been regarded as the standard for quick antidepressant response [8]. A study showed that ketamine is as effective as ECT in improving depressive symptoms in MDD patients, but ketamine had a faster onset and better antidepressant effects [9]. Moreover, ketamine helps improve neurocognitive function, especially attention and executive function, so it may be a more favorable short-term treatment option than ECT [10].

**Figure 1:**
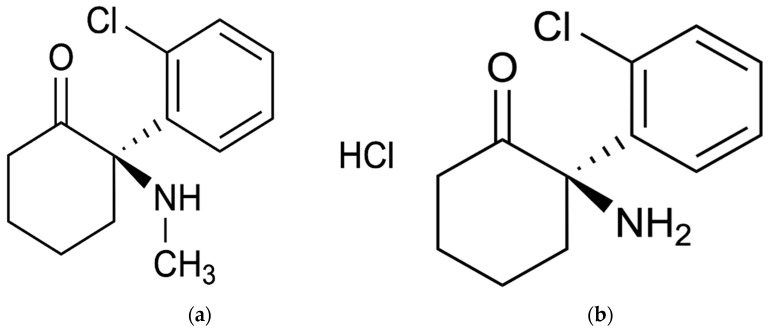
Chemical structures of (R)-ketamine hydrochloride (**a**) and (R)-norketamine (**b**).

Ketamine is a racemic mixture consisting of (R)-ketamine (or arketamine) and (S)-ketamine (or esketamine) [11], among which, (S)-ketamine was the first to be developed as an antidepressant and received the U.S. Food and Drug Administration (FDA) breakthrough therapy designation. As the N-methyl-D-aspartate receptor (NMDAR) antagonist, (S)-enantiomer has higher affinity than the racemate and (R)-enantiomer for the NMDAR [11]. However, according to pre-clinical studies, (R)-ketamine has greater potency and longer-lasting antidepressant actions, and significantly reduces psychotropic side effects [12,13]. In humans, when the injected dose of (R)-ketamine (1.8 mg/kg) was 4 times that of (S)-ketamine (0.45 mg/kg), only 39% of patients experienced psychotomimetic side effects such as hallucinations [14]. Furthermore, in a two-phase test of healthy subjects, it has been found that when given the equimolar doses of (S)-ketamine and (R)-ketamine, the plasma concentration levels of (S)- and (R)-ketamine ranged from 269 to 523 ng/mL (mean 379 ± 71 ng/mL) and from 280 to 508 ng/mL (mean 389 ± 74 ng/mL) between subjects. At the mean plasma concentration level of 539 ng/mL, (S)-ketamine might cause psychotomimetic reactions (such as the changes of auditory, visual, proprioceptive, dissociation and hallucinations), while (R)-ketamine did not show these reactions. In contrast, the feeling of relaxation was related to (R)-ketamine [15]. Taken together, in both animal models and human studies, (R)-ketamine showed fewer psychiatric side effects than ketamine and (S)-ketamine. Therefore, (R)-ketamine would be a better choice to relieve symptoms and reduce the risk of suicide in patients with TRD.

(R)-ketamine hydrochloride injection, chemically known as (2R)-2-(2-Chlorophenyl)-2-(methylamino) cyclohexanone hydrochloride (Figure 1), is a novel rapid-acting antidepressant for the treatment of TRD. Moreover, (R)-ketamine is mainly metabolized by hepatic cytochrome P450-mediated N-demethylation to (R)-norketamine [16], chemically known as (R)-2-amino-2-(2-Chlorophenyl) cyclohexanone (Figure 1).

Clinical pharmacokinetics (PK) describes the dynamic changes of drug absorption, distribution, metabolism and excretion (ADME) in the human body, which has great reference value for how to provide patients with a safe and effective drug dose design. Moreover, safety and tolerability assessments are also essential components of the new drug development process.

In 2019, Leal GC et al. conducted an open-label pilot trial that first investigated the efficacy and safety of a single intravenous infusion of (R)-ketamine for TRD in humans [17]. Then, in 2022, they further conducted a placebo-controlled pilot study to explore the feasibility of a randomized controlled trial of (R)-ketamine for TRD [18]. Both pilot studies had the limitation of small samples and were underpowered.

Recently, Jiangsu Nhwa Pharmaceutical Co., Ltd. has successfully synthesized (R)-ketamine hydrochloride and developed (R)-ketamine hydrochloride injection. In this study, we conducted a first-in-human (FIH) Phase I clinical trial to evaluate the safety and tolerability and pharmacokinetics profiles of the (R)-ketamine hydrochloride injection by ascending single intravenous doses ranging from 10.0 mg to 180 mg in healthy Chinese subjects.

## 2. Results

### 2.1. Study Population

A total of fifty subjects were enrolled in this trial: forty were dosed with (R)-ketamine hydrochloride injection and ten were dosed with placebo across five dose cohorts. The safety analysis set (SS) included all the randomized subjects. The pharmacokinetics set (PKS) included all subjects who received (R)-ketamine hydrochloride injection. The baseline demographic characteristics of the study population are summarized in Table 1. Among the 50 subjects, gender distribution was balanced with 50% male subjects and 50% female subjects; 49 subjects were of Han nationality and 1 subject was Mongolian. The average age of the subjects was 32.3 years (range: 19 to 44 years). The average height of the subjects was 164.31 cm (range: 148.5 to 179.5 cm). The mean baseline body weight was 62.37 kg (range: 47.2 to 83.3 kg). The mean body mass index (BMI) was 23.12 kg/m^2^ (range: 18.9 to 27.0 kg/m^2^).

**Table 1:** Baseline demographic data of the study population.

Characteristic	Dose Cohorts						
	10.0 mg	30.0 mg	60.0 mg	120 mg	180 mg	Placebo	Total
**n**	8	8	8	8	8	10	50
**Sex** n, Male/female	4/4	4/4	3/5	3/5	4/4	7/3	25/25
**Age, years**							
Mean (SD)	33.4 (4.5)	26.6 (8.2)	34.9 (7.3)	35.5 (6.4)	34.1 (5.7)	29.9 (7.5)	32.3 (7.1)
Max, Min	26, 41	20, 44	25, 44	25, 44	28, 42	19, 41	19, 44
Median	33.0	24.0	35.5	37.0	32.0	32.0	32.0
**Ethnic group** n, Han/others	8/0	8/0	7/1	8/0	8/0	10/0	49/1
**Height, cm**							
Mean (SD)	164.88 (6.95)	166.00 (5.32)	162.75 (5.81)	165.00 (6.77)	162.25 (5.71)	164.85 (10.65)	164.31 (7.04)
Max, Min	151.5, 174.0	160.0, 176.5	155.0, 171.0	158.5, 177.5	153.0, 168.5	148.5, 179.5	148.5, 179.5
**Weight, kg**							
Mean (SD)	64.70 (10.31)	57.93 (5.21)	61.65 (4.61)	64.58 (7.23)	59.71 (7.74)	64.99 (8.87)	62.37 (7.75)
Max, Min	47.2, 77.9	50.2, 65.3	54.6, 69.9	54.7, 77.2	49.1, 71.4	54.0, 83.3	47.2, 83.3
**BMI, kg/m^2^**							
Mean (SD)	23.76 (2.25)	21.06 (1.77)	23.31 (2.55)	23.83 (1.95)	22.58 (2.03)	23.98 (1.57)	23.12 (2.17)
Max, Min	20.3, 25.9	19.0, 24.9	20.5, 27.0	20.6, 26.5	18.9, 25.3	20.4, 25.5	18.9, 27.0

n, number of samples. BMI, body mass index; SD, standard deviation; Max, maximum; Min, minimum.

### 2.2. Pharmacokinetics

**Figure 2:**
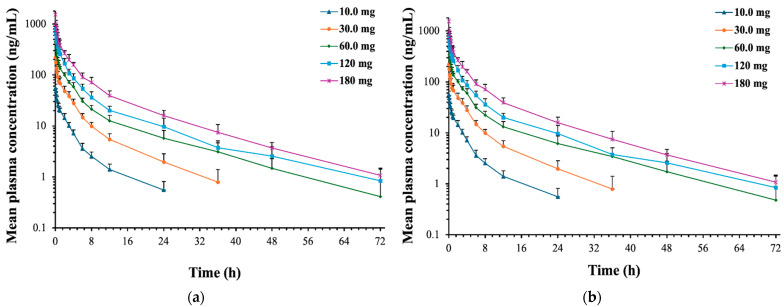
Mean plasma concentration–time curves of (R)-ketamine in healthy Chinese subjects after single intravenous administration (semilogarithmic scale). (**a**) n = 7, excluding subject 3-001. (**b**) n = 8, including subject 3-001. From the pre-dose screening period to the fourth day after the administration, the lymphocyte counts of the subject 3-001 decreased from 1.85 to 0.81 (normal value 1.1~3.2), and the association with the drug was assessed as possibly unrelated to the drug. Bars represent SDs.

**Figure 3:**
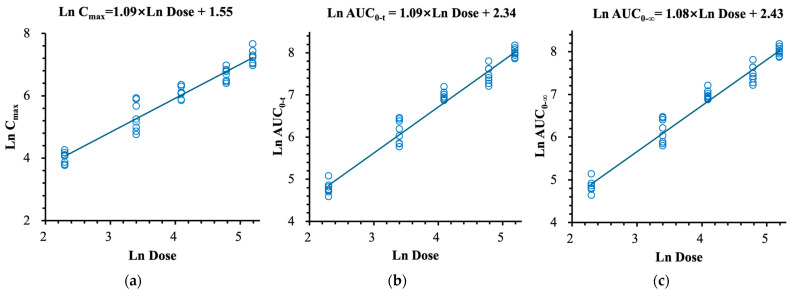
The relationships between PK parameters and dose of (R)-ketamine after single intravenous doses. (**a**) C_max_. (**b**) AUC_0–t_. (**c**) AUC_0–∞_.

**Figure 4:**
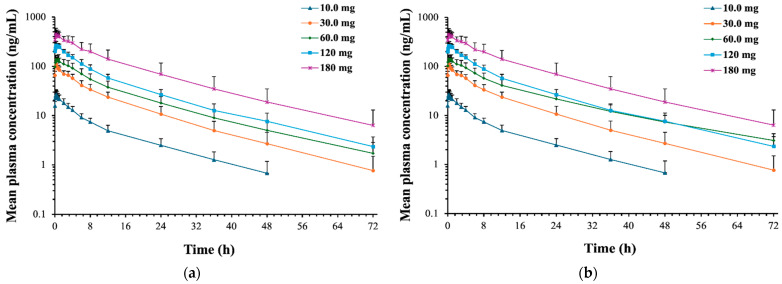
Mean plasma concentration–time curves of (R)-norketamine in healthy Chinese subjects after single intravenous administration (semilogarithmic scale). (**a**) n = 7, excluding subject 3-001. (**b**) n = 8, including subject 3-001. From the pre-dose screening period to the fourth day after the administration, the lymphocyte counts of the subject 3-001 decreased from 1.85 to 0.81 (normal value 1.1~3.2), and the association with the drug was assessed as possibly unrelated to the drug. Bars represent SDs.

**Figure 5:**
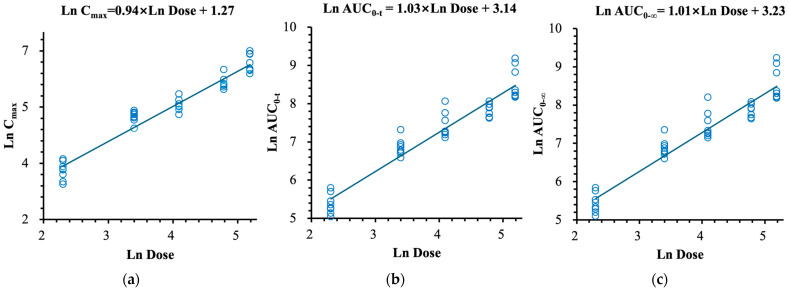
The relationships between PK parameters and dose of (R)-norketamine after single intravenous doses. (**a**) C_max_. (**b**) AUC_0–t_. (**c**) AUC_0–∞_.

The detailed pharmacokinetic parameters of (R)-ketamine and (R)-norketamine are summarized in Table 2 and Table 3, respectively. The mean plasma concentration–time profiles, the relationships between PK parameters and dose of (R)-ketamine are presented in Figure 2 and Figure 3, respectively. The mean plasma concentration–time profiles, the relationships between PK parameters and dose of (R)-norketamine are presented in Figure 4 and Figure 5, respectively.

#### 2.2.1. (R)-Ketamine

The mean C_max_, AUC_0–t_, and AUC_0–∞_ of (R)-ketamine in each dose cohort are shown in Table 2, which suggests that the exposure values of (R)-ketamine (C_max_ and AUC) increased proportionally with the dose. The relationships between the dose and exposure were assessed by the method combining the log-linearized power model and the confidence interval criterion. The log-linearized power can be described as ln(PK) = β_0_ + β_1_ × ln(Dose), where β_0_ is intercept and β_1_ is slope. The estimated slope values (β_1_) and 95% confidence intervals for the C_max_, AUC_0–t_, and AUC_0–∞_ calculated by the power model were 1.09 (1.01, 1.18), 1.09 (1.03, 1.16) and 1.08 (1.01, 1.14), respectively, which all approached 1.000, see Figure 3. Moreover, the 95% confidence intervals of the β_1_ values overlapped with the judgment intervals (0.92, 1.08), which indicated the linear pharmacokinetic characteristics of (R)-ketamine over the intravenous dose range of 10.0~180 mg (R)-ketamine hydrochloride.

**Table 2:** Main pharmacokinetic parameters of the parent drug (R)-ketamine in healthy Chinese subjects after single intravenous doses.

Dose Cohorts	C_max_ (ng/mL)	T_max_ (h)	AUC_0–t_ (h·ng/mL)	AUC_0–∞_ (h·ng/mL)	CL (L/h)	V_d_ (L)	λ_z_ (/h)	t_1/2_ (h)	MRT_0–t_ (h)	MRT_0–∞_ (h)	AUMC_0–t_ (h^2^·ng/mL)	AUMC_0–∞_ (h^2^·ng/mL)
10.0 mg (n = 8)	56.0 (9.9, 17.7)	0.67 (0.67–0.67)	121 (18, 15.3)	129 (21, 16.3)	79.2 (11.6, 14.6)	817 (209, 25.5)	0.104 (0.040, 38.4)	7.19 (1.63, 22.6)	3.97 (0.63, 15.8)	5.46 (1.06, 19.4)	432 (117, 27.0)	688 (209, 30.5)
30.0 mg (n = 8)	222 (104, 46.9)	0.67 (0.66–0.75)	468 (131, 28.0)	478 (134, 28.0)	67.3 (18.6, 27.7)	828 (234, 28.3)	0.0882 (0.0426, 48.3)	9.11 (3.07, 33.7)	5.27 (0.85, 16.1)	6.26 (1.21, 19.3)	2337 (879, 37.6)	2888 (1170, 40.5)
60.0 mg (n = 7)	448 (85, 19.0)	0.67 (0.67–0.67)	1031 (75, 7.3)	1047 (76, 7.2)	57.5 (4.0, 6.9)	1066 (251, 23.6)	0.0573 (0.0180, 31.4)	12.9 (3.1, 23.9)	7.48 (2.25, 30.1)	8.55 (2.47, 28.8)	7411 (2661, 35.9)	8659 (2912, 33.6)
* 60.0 mg (n = 8, including subject 3-001)	463 (90, 19.4)	0.67 (0.67–0.67)	1069 (126, 11.8)	1086 (127, 11.7)	55.9 (5.9, 10.6)	1050 (237, 22.5)	0.0560 (0.0170, 30.4)	13.1 (2.9, 22.2)	7.78 (2.25, 29.0)	8.89 (2.47, 27.8)	8070 (3090, 38.3)	9398 (3410, 36.3)
120 mg (n = 8)	805 (166, 20.6)	0.67 (0.67–0.75)	1774 (364, 20.5)	1815 (392, 21.6)	68.7 (14.4, 20.9)	1327 (285, 21.5)	0.0529 (0.0115, 21.8)	13.6 (2.7, 20.0)	7.45 (1.44, 19,3)	8.16 (1.53, 18.7)	12,955 (4474, 34.5)	14,849 (4957, 33.4)
180 mg (n = 8)	1424 (350, 24.6)	0.67 (0.67–0.68)	2997 (333, 11.1)	3018 (331, 11.0)	60.3 (6.5, 10.7)	1185 (304, 25.7)	0.0527 (0.0099, 18.8)	13.6 (2.7, 19.6)	7.38 (1.36, 18.4)	8.00 (1.52, 18.9)	20,974 (4087, 19.5)	22,953 (4434, 19.3)

n, number of samples. C_max_, maximum drug concentration in plasma; T_max_, the time to reach C_max_; AUC_0–t_, area under the concentration–time curve from zero to the last measurable concentration; AUC_0–∞_, area under the concentration–time curve from zero to infinity; CL, clearance; V_d_, Volume of distribution; λ_z_, elimination rate constant; t_1/2_, elimination half-life; MRT_0–t_, mean residence time from zero to the last measurable concentration; MRT_0–∞_, mean residence time from time zero to infinity; AUMC_0–t_, area under the moment curve from zero to the last measurable concentration; AUMC_0–∞_, area under the moment curve from zero to infinity. All values are expressed as mean (SD, CV%), except for T_max_ values, which are expressed as median (minimum, maximum). * From the pre-dose screening period to the fourth day after the administration, the lymphocyte counts of the subject 3-001 decreased from 1.85 to 0.81 (normal value 1.1~3.2), and the association with the drug was assessed as possibly unrelated to the drug.

#### 2.2.2. (R)-Norketamine

After a single intravenous infusion of 10.0~180 mg (R)-ketamine hydrochloride injection in healthy subjects, it was found that the half-life of (R)-norketamine in the 60.0 mg dose cohort was prolonged, see Table 3 and Figure 4b. This phenomenon was caused by the prolonged (R)-norketamine half-life of 25.4 h for one subject (subject 3-001). The prolonged half-life in subject 3-001 might be caused by the treatment-emergent adverse event (TEAE). From the pre-dose screening period to the fourth day after the drug administration, the lymphocyte counts of the subject 3-001 decreased from 1.85 to 0.81 (normal value 1.1~3.2). This TEAE was assessed by the clinical doctor as possibly unrelated to (R)-ketamine hydrochloride injection. Although a prolonged half-life appeared in subject 3-001, the exposure of (R)-norketamine still increased proportionally with the 60.0 mg dose in the cohort.

In each dose cohort, the mean MPratio_C_max_ and MPratio_AUC of metabolite (R)-norketamine relative to the parent drug (R)-ketamine were 0.375~0.642 and 1.53~2.31, respectively. Although the C_max_ of (R)-norketamine was lower than (R)-ketamine, the total exposure to the metabolite (R)-norketamine was still higher than the parent drug (R)-ketamine. The estimated β_1_ values and 95% confidence intervals for C_max_, AUC_0–t_, and AUC_0–∞_ calculated by the power model were 0.94 (0.86, 1.01), 1.03 (0.93, 1.13) and 1.01 (0.91, 1.12), respectively, which all approached 1.000, see Figure 5. The 95% confidence intervals of β_1_ values for both C_max_ and AUC included the value of 1.000, which indicated the linear pharmacokinetic characteristics of (R)-norketamine over the intravenous dose range of 10.0~180 mg (R)-ketamine hydrochloride.

**Table 3:** Main pharmacokinetic parameters of the metabolite (R)-norketamine in healthy Chinese subjects after single intravenous doses.

Dose Cohorts	C_max_ (ng/mL)	T_max_ (h)	AUC_0–t_ (h·ng/mL)	AUC_0–∞_ (h·ng/mL)	λ_z_ (h)	t_1/2_ (h)	MRT_0–t_ (h)	MRT_0–∞_ (h)	AUMC_0–t_ (h^2^·ng/mL)	AUMC_0–∞_ (h^2^·ng/mL)	MPratio C_max_	MPratio_AUC_0–∞_
10.0 mg (n = 8)	27.7 (6.5, 23.5)	0.92 (0.75–1.34)	222 (61, 27.4)	236 (64, 26.9)	0.0524 (0.0162, 30.9)	14.3 (4.2, 29.6)	12.0 (2.9, 23.7)	15.6 (4.0, 25.5)	2674 (1365, 51.1)	3739 (1872, 50.1)	0.534 (0.124, 23.2)	1.95 (0.46, 23.8)
30.0 mg (n = 8)	115 (16, 13.9)	0.92 (0.75–1.17)	972 (239, 24.5)	990 (249, 25.1)	0.0636 (0.0234, 36.9)	12.0 (3.6, 29.8)	12.9 (2.7, 20.9)	14.2 (3.5, 24.5)	12,457 (5379, 43.2)	14,080 (6673, 47.4)	0.642 (0.241, 37.6)	2.31 (0.63, 27.3)
60.0 mg (n = 7)	153 (28, 18.4)	1.19 (0.83–1.50)	1574 (395, 25.1)	1617 (407, 25.2)	0.0535 (0.0179, 33.4)	13.8 (3.1, 22.5)	14.0 (2.9, 20.8)	15.8 (4.0, 25.2)	21,700 (8315, 38.3)	25,365 (10356, 40.8)	0.375 (0.113, 30.2)	1.65 (0.43, 26.4)
* 60.0mg (n = 8, including subject 3-001)	156 (28, 17.7)	1.13 (0.83–1.50)	1775 (673, 37.9)	1868 (810, 43.3)	0.0503 (0.0190, 37.7)	15.3 (5.0, 32.7)	15.1 (4.2, 27.7)	18.1 (7.4, 41.1)	27,870 (19,074, 68.4)	37,461 (35,530, 94.8)	0.369 (0.106, 28.7)	1.80 (0.59, 32.8)
120 mg (n = 8)	285 (51, 17.9)	0.92 (0.67–1.50)	2566 (397, 15.5)	2615 (418, 16.0)	0.0518 (0.0084, 16.2)	13.7 (2.0, 14.9)	13.1 (2.0, 15.0)	14.5 (2.8, 19.4)	32,860 (8898, 27.1)	37,472 (12,006, 32.0)	0.392 (0.110, 27.9)	1.53 (0.35, 22.6)
180 mg (n = 8)	491 (119, 24.3)	1.08 (0.83–4.67)	5506 (2495, 45.3)	5643 (2646, 46.9)	0.0523 (0.0079, 15.1)	13.5 (2.1, 15.3)	14.1 (3.0, 21.5)	15.6 (4.0, 25.4)	81,382 (54,954, 67.5)	94,261 (69,617, 73.9)	0.380 (0.117, 30.8)	1.98 (0.85, 42.8)

n, number of samples. C_max_, maximum drug concentration in plasma; T_max_, the time to reach C_max_; AUC_0–t_, area under the concentration–time curve from zero to the last measurable concentration; AUC_0–∞_, area under the concentration–time curve from zero to infinity; λ_z_, elimination rate constant; t_1/2_, elimination half-life; MRT_0–t_, mean residence time from zero to the last measurable concentration; MRT_0–∞_, mean residence time from time zero to infinity; AUMC_0–t_, area under the moment curve from zero to the last measurable concentration; AUMC_0–∞_, area under the moment curve from zero to infinity; MPratio_C_max_, the ratio of C_max_ of the metabolite to the parent drug; MPratio_AUC_0–∞_, the ratio of AUC_0–∞_ of the metabolite to the parent drug. All values are expressed as mean (SD, CV%), except for T_max_ values, which are expressed as median (minimum, maximum). * From the pre-dose screening period to the fourth day after the administration, the lymphocyte counts of the subject 3-001 decreased from 1.85 to 0.81 (normal value 1.1~3.2), and the association with the drug was assessed as possibly unrelated.

### 2.3. Safety and Tolerability

The detailed adverse events of (R)-ketamine hydrochloride injection are summarized in Table 4. Among the 40 subjects administered (R)-ketamine hydrochloride injection in the five dose cohorts, 35 subjects (87.5%) had 119 cases of TEAE. One case was moderate in severity, which occurred on the reporting day in a subject in the 180 mg dose cohort. Except for this case, the remaining 118 cases were mild. Only one mild TEAE (the symptom was elevated serum phosphorus) in the 30.0 mg dose cohort was converted to unknown because the subject refused a return visit. The remaining TEAE symptoms all resolved spontaneously without treatment. In addition, among the 10 subjects in the placebo group, 4 subjects (40.0%) had 4 cases of TEAE, all of which were mild and recovered spontaneously without treatment. Among the 40 subjects treated with (R)-ketamine hydrochloride injection in the trial, 32 subjects (80.0%) had 107 cases of adverse drug reaction (ADR). Except for 1 case (the symptom was somnolence, which was moderate), the remaining 106 cases were mild, and among the 10 subjects in the placebo group, 2 subjects (20.0%) had 2 cases of ADR, both of which were mild. There was no TEAE, serious adverse event (SAE), or death leading to study termination. In the 10.0 mg dose cohort, the incidence of TEAE and ADR occurred in the subjects treated with (R)-ketamine hydrochloride injection was comparable to those treated with the placebo. In the whole trial, the incidence of TEAE and ADR in the subjects treated with (R)-ketamine hydrochloride injection was significantly higher than those treated with the placebo. Among all TEAEs and ADRs, the symptoms of dizziness, somnolence and dissociation showed a dose-dependent increase trend, see Table 5 and Table 6. Overall, in the dose range of 10.0~180 mg, a single 40 min fasting intravenous infusion of (R)-ketamine hydrochloride injection is safe and well tolerated in healthy subjects.

**Table 4:** Summary of adverse events of (R)-ketamine hydrochloride injection.

Adverse Event	Dose Cohorts						
	10.0 mg	30.0 mg	60.0 mg	120 mg	180 mg	Placebo	Total (No Placebo)
n	8	8	8	8	8	10	40
TEAE							
mild	3 (37.5%) [6]	7 (87.5%) [21]	8 (100.0%) [32]	8 (100.0%) [22]	8 (100.0%) [37]	4 (40.0%) [4]	34 (85.0%) [118]
moderate	0	0	0	0	1 (12.5%) [1]	0	1 (2.5%) [1]
severe	0	0	0	0	0	0	0
ADR							
mild	2 (25.0%) [4]	7 (87.5%) [18]	7 (87.5%) [30]	7 (87.5%) [18]	8 (100.0%) [36]	2 (20.0%) [2]	31 (77.5%) [106]
moderate	0	0	0	0	1 (12.5%) [1]	0	1 (2.5%) [1]
severe	0	0	0	0	0	0	0
SAE							
Suspicious and unexpected severe ADRs	0	0	0	0	0	0	0
TEAEs leading to study termination	0	0	0	0	0	0	0
ADRs leading to study termination	0	0	0	0	0	0	0
Severe ADRs leading to study termination	0	0	0	0	0	0	0
SAEs leading to study termination	0	0	0	0	0	0	0

n, number of samples. The values are presented as a number (percentage) [number of cases]. TEAE, treatment-emergent adverse event; ADR, adverse drug reaction; SAE, serious adverse event. The placebo cohort was not included in the safety analysis set.

**Table 5:** Summary of TEAEs of (R)-ketamine hydrochloride injection.

TEAEs	Dose Cohorts						
	10.0 mg (n = 8)	30.0 mg (n = 8)	60.0 mg (n = 8)	120 mg (n = 8)	180 mg (n = 8)	Placebo (n = 10)	Total (No Placebo) (n = 40)
Dizziness	0	5 (62.5%) [5]	5 (62.5%) [6]	5 (62.5%) [5]	6 (75.0%) [6]	0	21 (52.5%) [22]
Somnolence	0	0	0	0	8 (100.0%) [9]	0	8 (20.0%) [9]
Decreased sensation	0	1 (12.5%) [1]	2 (25.0%) [2]	1 (12.5%) [1]	0	0	4 (10.0%) [4]
Decreased level of consciousness	0	1 (12.5%) [1]	0	0	1 (12.5%) [1]	1 (10.0%) [1]	2 (5.0%) [2]
Sleepiness	0	1 (12.5%) [1]	0	0	1 (12.5%) [1]	0	2 (5.0%) [2]
Hypertonia	0	0	0	0	1 (12.5%) [1]	0	1 (2.5%) [1]
Head discomfort	0	0	1 (12.5%) [1]	0	0	0	1 (2.5%) [1]
Headache	0	0	1 (12.5%) [1]	0	0	0	1 (2.5%) [1]
Tremor	0	0	1 (12.5%) [1]	0	0	0	1 (2.5%) [1]
Blood pressure reduction	1 (12.5%) [2]	2 (25.0%) [2]	1 (12.5%) [1]	0	0	0	4 (10.0%) [5]
Serum bilirubin elevation	0	0	0	0	2 (25.0%) [2]	1 (10.0%) [1]	2 (5.0%) [2]
Positive urine leukocytes	0	1 (12.5%) [1]	0	1 (12.5%) [1]	0	0	2 (5.0%) [2]
Blood glucose elevation	0	0	0	1 (12.5%) [1]	0	1 (10.0%) [1]	1 (2.5%) [1]
Decreased neutrophil count	0	0	0	1 (12.5%) [1]	1 (12.5%) [1]	0	2 (5.0%) [2]
Decreased lymphocyte count	0	0	1 (12.5%) [1]	0	0	0	1 (2.5%) [1]
Decreased heart rate	0	0	1 (12.5%) [2]	0	0	0	1 (2.5%) [2]
Serum phosphorus elevation	0	1 (12.5%) [1]	0	0	0	0	1 (2.5%) [1]
Blood pressure elevation	1 (12.5%) [1]	0	0	0	0	0	1 (2.5%) [1]
Dissociation	0	1 (12.5%) [1]	3 (37.5%) [3]	3 (37.5%) [3]	8 (100.0%) [8]	0	15 (37.5%) [15]
Derealization	0	0	0	1 (12.5%) [1]	0	0	1 (2.5%) [1]
Drunkenness	0	1 (12.5%) [1]	5 (62.5%) [5]	2 (25.0%) [2]	1 (12.5%) [1]	0	9 (22.5%) [9]
Fatigue	0	0	0	0	1 (12.5%) [1]	0	1 (2.5%) [1]
Feel hot	0	1 (12.5%) [1]	0	0	0	0	1 (2.5%) [1]
Thirst	0	0	0	0	1 (12.5%) [1]	0	1 (2.5%) [1]
Nausea	0	2 (25.0%) [2]	1 (12.5%) [1]	2 (25.0%) [2]	2 (25.0%) [2]	0	7 (17.5%) [7]
Vomiting	0	2 (25.0%) [2]	0	2 (25.0%) [2]	0	0	4 (10.0%) [4]
Dry mouth	0	0	3 (37.5%) [3]	0	0	0	3 (7.5%) [3]
Blurred vision	0	0	2 (25.0%) [2]	0	3 (37.5%) [3]	0	5 (12.5%) [5]
Sinus bradycardia	1 (12.5%) [1]	1 (12.5%) [1]	2 (25.0%) [2]	0	0	0	4 (10.0%) [4]
Anemia	1 (12.5%) [1]	0	0	0	1 (12.5%) [1]	1 (10.0%) [1]	2 (5.0%) [2]
Urinary tract infections	1 (12.5%) [1]	0	0	1 (12.5%) [1]	0	0	2 (5.0%) [2]
Excessive sweating	0	0	0	2 (25.0%) [2]	0	0	2 (5.0%) [2]
Dry nose	0	0	1 (12.5%) [1]	0	0	0	1 (2.5%) [1]
Hematuria	0	1 (12.5%) [1]	0	0	0	0	1 (2.5%) [1]

n, number of samples. The values are presented as number (percentage) [number of cases]. TEAE, treatment-emergent adverse event. The placebo cohort was not included in the safety analysis set.

**Table 6:** Summary of ADRs of (R)-ketamine hydrochloride injection.

ADRs	Dose Cohorts						
	10.0 mg (n = 8)	30.0 mg (n = 8)	60.0 mg (n = 8)	120 mg (n = 8)	180 mg (n = 8)	Placebo (n = 10)	Total (No Placebo) (n = 40)
Dizziness	0	5 (62.5%) [5]	5 (62.5%) [6]	5 (62.5%) [5]	6 (75.0%) [6]	0	21 (52.5%) [22]
Somnolence	0	0	0	0	8 (100.0%) [9]	0	8 (20.0%) [9]
Decreased sensation	0	1 (12.5%) [1]	2 (25.0%) [2]	1 (12.5%) [1]	0	0	4 (10.0%) [4]
Decreased level of consciousness	0	1 (12.5%) [1]	0	0	1 (12.5%) [1]	1 (10.0%) [1]	2 (5.0%) [2]
Sleepiness	0	1 (12.5%) [1]	0	0	1 (12.5%) [1]	0	2 (5.0%) [2]
Hypertonia	0	0	0	0	1 (12.5%) [1]	0	1 (2.5%) [1]
Head discomfort	0	0	1 (12.5%) [1]	0	0	0	1 (2.5%) [1]
Headache	0	0	1 (12.5%) [1]	0	0	0	1 (2.5%) [1]
Tremor	0	0	1 (12.5%) [1]	0	0	0	1 (2.5%) [1]
Dissociation	0	1 (12.5%) [1]	3 (37.5%) [3]	3 (37.5%) [3]	8 (100.0%) [8]	0	15 (37.5%) [15]
Derealization	0	0	0	1 (12.5%) [1]	0	0	1 (2.5%) [1]
Drunkenness	0	1 (12.5%) [1]	5 (62.5%) [5]	2 (25.0%) [2]	1 (12.5%) [1]	0	9 (22.5%) [9]
Fatigue	0	0	0	0	1 (12.5%) [1]	0	1 (2.5%) [1]
Feel hot	0	1 (12.5%) [1]	0	0	0	0	1 (2.5%) [1]
Thirst	0	0	0	0	1 (12.5%) [1]	0	1 (2.5%) [1]
Nausea	0	2 (25.0%) [2]	1 (12.5%) [1]	2 (25.0%) [2]	2 (25.0%) [2]	0	7 (17.5%) [7]
Vomiting	0	2 (25.0%) [2]	0	2 (25.0%) [2]	0	0	4 (10.0%) [4]
Dry mouth	0	0	3 (37.5%) [3]	0	0	0	3 (7.5%) [3]
Blood pressure reduction	1 (12.5%) [2]	2 (25.0%) [2]	1 (12.5%) [1]	0	0	0	4 (10.0%) [5]
Serum bilirubin elevation	0	0	0	0	2 (25.0%) [2]	1 (10.0%) [1]	2 (5.0%) [2]
Decreased heart rate	0	0	1 (12.5%) [2]	0	0	0	1 (2.5%) [2]
Blood pressure elevation	1 (12.5%) [1]	0	0	0	0	0	1 (2.5%) [1]
Decreased neutrophil count	0	0	0	0	1 (12.5%) [1]	0	1 (2.5%) [1]
Blurred vision	0	0	2 (25.0%) [2]	0	3 (37.5%) [3]	0	5 (12.5%) [5]
sinus bradycardia	1 (12.5%) [1]	1 (12.5%) [1]	1 (12.5%) [1]	0	0	0	3 (7.5%) [3]
Excessive sweating	0	0	0	2 (25.0%) [2]	0	0	2 (5.0%) [2]
Dry nose	0	0	1 (12.5%) [1]	0	0	0	1 (2.5%) [1]

n, number of samples. The values are presented as number (percentage) [number of cases]. ADR, adverse drug reaction. The placebo cohort was not included in the safety analysis set.

## 3. Discussion

### 3.1. Safety and Tolerability

The trial results showed that (R)-ketamine hydrochloride injection was safe and well tolerated throughout the study. Overall, the TEAEs and ADRs were generally mild and recovered spontaneously without treatment, and the symptoms of dizziness, somnolence and dissociation showed a dose-dependent increase trend. The single doses of (R)-ketamine hydrochloride did not cause serious adverse events or discontinuations. The TEAEs with high incidence were associated with neurological adverse effects of NMDA receptor inhibitors. According to previous research, after intravenous ketamine or (S)-ketamine, about half of the patients experienced adverse effects of dissociation and nausea, and these adverse effects were too intense for some of them, so they had to be alleviated with dose reductions or medical treatment [19]. In this trial, the TEAEs with high incidence in the 40 subjects administered (R)-ketamine hydrochloride injection in the five dose cohorts were dizziness (21/40), dissociation (15/40), drunkenness (9/40), and somnolence (8/40); these subjects then recovered spontaneously. Other TEAEs were also resolved at the end of the trial without any medical treatment. The overall data indicated that (R)-ketamine hydrochloride injection has a favorable safety and tolerability, which can also support further clinical studies in patients.

### 3.2. Dose Range Design

The dose range used in this single ascending dose (SAD) study was determined based on the safety, tolerability and efficacy data obtained from preclinical studies of (R)-ketamine hydrochloride, and human maximal tolerance dose (MTD) was explored as much as possible under the premise of ensuring the safety of subjects.

#### 3.2.1. Starting Dose Design

To predict the clinical pharmacokinetics of (R)-ketamine hydrochloride in humans, a series of in vitro and in vivo pharmacokinetic data from animals can be used. Currently, there are four main methods and models used to predict clinical pharmacokinetics: in vitro–in vivo extrapolation (IVIVE) [20], using in vivo data from animals for allometric scaling (AS) [21], physiologically based pharmacokinetic modeling [22], and combining mechanistic modeling with machine learning [23].

The starting dose of (R)-ketamine hydrochloride was determined based on the in vitro metabolism, in vivo PK and safety data, and also according to NMPA guidance for estimating the maximum recommended starting dose (MRSD) of drugs in the first clinical trial of healthy adult volunteers [24] and FDA guidance for estimating the maximum safe starting dose in initial clinical trials for therapeutics in adult healthy volunteers [25].

In general, no observed adverse effect level (NOAEL) of multiple species achieved in preclinical toxicology studies is required to estimate MRSD. After obtaining the NOAELs of multiple species, they were converted to human equivalent doses (HEDs) based on the method of body surface area normalization. The species with the lowest HED were considered to be the most suitable species used to derive the MRSD for healthy adult subjects. Then, the lowest HED was divided by a safety factor (SF) to obtain the MRSD. According to the NMPA and FDA guidelines, the safety factor was usually selected as 10. The safety factor is to ensure that the first dose in humans would not cause adverse effects.

On the basis of the results of preclinical in vitro liver microsomal metabolic stability study and in vivo PK study in animals of (R)-ketamine hydrochloride, the mean value of human intravenous clearance (CL) was 193.4 L/h by using IVIVE, single species AS method and double species AS method. The NOAEL for male and female SD rats obtained from a preclinical toxicology study (administered once daily for 4 weeks) was 15.0 mg/kg, and the HED was 144 mg. In order to further assure the safety of subjects, the safety factor was selected as 10, and the body weight was calculated as 60 kg, so the MRSD was 14.4 mg. After 4 weeks of continuous administration in SD rats, the AUC_0–t_ of female and male SD rats at the NOAEL dose was 2907.931 h·ng/mL and 2079.141 h·ng/mL, respectively. Assuming that the human body reached the same exposure as that of SD rats, the HEDs were 562.39 mg and 402.11 mg calculated from the predicted CL of human intravenous administration, respectively, and the MRSDs were 56.24 mg and 40.21 mg after correction by SF.

According to the preclinical toxicology study (administered once daily for 4 weeks) in *beagles*, the NOAEL in female and male *beagles* was 20.0 mg/kg. The HED was calculated as 648 mg by the body surface area normalization method. After SF correction, the MRSD was 64.8 mg based on the body weight of 60 kg. According to the toxicokinetic study that accompanied the long-term toxicological study, after 4 weeks of continuous administration, the AUC_0–t_ of female and male *beagles* at the NOAEL dose was 3296.135 h·ng/mL and 3954.165 h·ng/mL, respectively. The HEDs were 637.47 mg and 764.74 mg calculated from the predicted CL of human intravenous administration, and the MRSDs were 63.75 mg and 76.47 mg; the data are shown in Table 7.

Based on the MRSD calculated from the above toxicological studies, the lowest dose was 14.4 mg. In this single-dose human trial, the starting dose was set at 10.0 mg to maximize the safety of the subjects and to allow for the convenience of clinical operability.

**Table 7:** NOAEL and MRSD of (R)-ketamine hydrochloride for 4-week-long toxicity experiments in preclinical studies.

Species	Sex	NOAEL (mg/kg/day)	HED ^1^ (mg)	AUC_0–t_ (h·ng/mL)	Predicted Human CL (L/h)	HED ^2^(mg)	SF	MRSD (mg)
SD rats	female/male	15.0	144	NA	NA	NA	10	14.4
*Beagle*	female/male	20.0	648	NA	NA	NA		64.8
SD rats	female	15.0	NA	2907.931	193.4	562.39		56.24
	male		NA	2079.141		402.11		40.21
*Beagle*	female	20.0	NA	3296.135		637.47		63.75
	male		NA	3954.165		764.74		76.47

NOAEL, no observed adverse effect level; HED, human equivalent dose; SF, safety factor; AUC_0−t_, area under the concentration–time curve from zero to the last measurable concentration; CL, clearance; MRSD, maximum recommended starting dose; NA, not applicable. ^1^ The HED was calculated based on the method of body surface area normalization and body weight was calculated as 60 kg. ^2^ According to the results of preclinical in vitro liver microsomal metabolic stability study and in vivo PK study in animals, the mean value of human intravenous clearance was predicted by IVIVE, single species AS and double species AS. The HED was calculated based on the mean value of human intravenous clearance and exposure.

#### 3.2.2. Ascending Dose Design

Preclinical toxicology studies have shown that the minimum HED was calculated as 402.11 mg based on the predicted CL of human intravenous administration (calculated from the data of male SD rats). Therefore, the MTD of a single dose in a human study should not exceed 402.11 mg; that is, the maximum ascending dose set in this study should be less than this HED. Considering the possible differences between animals and humans and the convenience of clinical research operation, 180 mg was selected as the maximum ascending dose to ensure the safety of subjects.

Preclinical pharmacodynamic studies showed that 0.5 h after administration of 20.0 and 40.0 mg/kg of (R)-ketamine hydrochloride, the immobility time of tail suspension in mice could be reduced, and the effect lasted for more than 24 h. A single intravenous injection of 10.0 and 20.0 mg/kg of (R)-ketamine hydrochloride for 0.5 h could shorten the immobility time of forced swimming in rats, but after 24 h, only the dose of 20.0 mg/kg had a significant effect. (R)-ketamine hydrochloride was administered intravenously at 5.0 and 10.0 mg/kg every other day (within a week, a total of four times), which can reduce the number of escape failures of rats in the learned helplessness experiment and has an antidepressant effect. Injection of 1.25 mg/kg and 2.5 mg/kg every other day (three times a week, a total of five weeks) can improve the problem of weight gain slowly in rats exposed to chronic unpredictable mild stress (CUMS), and injection of 5.0 mg/kg and 2.5 mg/kg every other day can improve the symptoms of reduced sucrose preference in CUMS rats.

According to the results of preclinical pharmacodynamic studies, the potential effective dose in humans may be 12.0~96.0 mg by the body surface area normalization. The maximum ascending dose chosen to explore in this study is higher than the potential effective dose in humans, which can ensure adequate exploration of the safety window and therapeutic window in future clinical research of (R)-ketamine hydrochloride. In addition, it should be noted that all the above calculations were based on internationally recognized empirical estimation methods, and there is no accurate theory to support them.

In summary, this clinical trial was started with a dose of 10.0 mg, and the dose was gradually increased until 180 mg. The investigators and the sponsor jointly decided whether to continue dose escalation after review of the safety data of each dose cohort. If the safety data did not reach the stopping criteria after administration of the maximum dose cohort, the decision to discontinue dose escalation or to add ascending dose cohorts would be discussed by the investigator and the sponsor.

### 3.3. Pharmacokinetics

After the intravenous administration of (R)-ketamine hydrochloride injection in healthy subjects, though the obtained C_max_ of the metabolite (R)-norketamine was lower than that of the parent drug (R)-ketamine (MPratio_C_max_ = 0.375~0.642), the total exposure to the metabolite was higher than that of the parent drug (MPratio_AUC = 1.53~2.31), which suggests that the main elimination pathway of (R)-ketamine hydrochloride in human may be metabolic elimination. After the intravenous doses administered from 10.0 mg to 180 mg, the C_max_ and AUC for both (R)-ketamine and its metabolite (R)-norketamine in the subjects increased approximately proportionally to the dose. These PK results support the development of a safe and effective therapeutic dose in the further clinical investigation of (R)-ketamine hydrochloride injection.

## 4. Materials and Methods

### 4.1. Chemicals and Materials

(R)-ketamine hydrochloride injection, placebo and the standard material were provided by Jiangsu Nhwa Pharmaceutical Co., Ltd. (Xuzhou, China). LC-grade acetonitrile, methanol and isopropanol were purchased from Sigma-Aldrich (St. Louis, MO, USA). Ammonium acetate (NH_4_Ac) and formic acid (FA) of ACS-grade were obtained from Sinopharm Chemical Reagent Co., Ltd. (Shanghai, China). Dimethyl sulfoxide (DMSO) of LC-grade was acquired from Adamas-beta (Basel, Switzerland). LC-grade ammonium hydroxide was from Aladdin (Beijing, China). Ultrapure water was generated by a Milli-Q Advantage A10 system (Merck Millipore, Darmstadt, Germany).

### 4.2. Study Population

The sponsor Jiangsu Nhwa Pharmaceutical Co., Ltd. plans to register (R)-ketamine hydrochloride injection in China, so the trial was only carried out in the Chinese mainland, and fifty Chinese healthy subjects (1:1 male to female ratio) were included. According to the inclusion and exclusion criteria, subjects were screened within 14 days prior to dosing. The eligible subjects were assigned a number at the time of enrollment. The numbering method was as follows: X-001 to X-010 for the ten subjects in every dose cohort, where X was the dose cohort number. There were five dose cohorts (10.0, 30.0, 60.0, 120 and 180 mg), so the dose cohort number X, named from 1 to 5, represented dose cohorts 10.0, 30.0, 60.0, 120 and 180 mg, respectively. For example, the ten subject codes from 1-001 to 1-010 were given for the ten subjects in the 10.0 mg dose cohort, 3-001 to 3-010 for the ten subjects in the 60.0 mg dose cohort, and so on. The subjects included in this trial were healthy men and women (non-lactating and nonpregnant) aged 18 to 45 with a body mass index (BMI) of 18.5~28.0 kg/m^2^, and the weight of male subjects was not less than 50 kg, while in the female subjects, it was not less than 45 kg. All subjects voluntarily participated in the trial and signed an informed consent form (ICF).

Exclusion criteria for subjects were as follows: (1) present illness history or past history of diseases or dysfunction affecting the clinical trial, including but not limited to diseases of the central nervous system, cardiovascular system, respiratory system, digestive system, urinary system, endocrine system, blood system, etc.; (2) present illness history or past history of mental disorder or brain dysfunction; (3) any surgical condition or condition that may significantly affect the absorption, distribution, metabolism and excretion of the drugs, or that may pose a hazard to the subjects enrolled in the trial, such as history of gastrointestinal surgery, urinary obstruction or dysuria, gastroenteritis, gastrointestinal ulcer etc.; (4) use of any drugs that inhibit or induce hepatic drug enzymes within the previous month; (5) had taken any medication within 2 weeks before the dose, including prescription and over-the-counter (OTC) medications; (6) be allergic to any component of the investigational drug, or have two or more drugs and food allergies; (7) vital signs, laboratory and electrocardiogram (ECG) findings were abnormal (e.g., corrected Q-T interval (QTc) > 450ms in men; QTc > 470ms in women, QTc interval was calculated using Fridericia’s formula); (8) non-negative for hepatitis B surface antigen (HBsAg), hepatitis C antibody (HCV-Ab), HIV antibody (HIV-Ab) and *treponema pallidum* antibody (TP-Ab); (9) had a history of alcohol abuse (i.e., more than 14 standard units per week (1 unit = 360 mL of beer or 45 mL of 40% spirits or 150 mL of wine) in the previous year or a breath test for alcohol was positive; (10) smoking more than five cigarettes per day on average in the previous 3 months or tobacco test was positive; (11) had a history of drug abuse or a positive urine drug screening test within the previous year; (12) refuse to use an effective nonpharmacologic contraceptive, or have a sperm or ovum donor plan; or a positive blood pregnancy test throughout the trial and for 3 months after the trial; (13) participated in any clinical trial within 3 months before dosing; (14) have special food requirements, cannot follow a uniform diet or have swallowing difficulties; (15) donated blood (including component donation) or massive blood loss (≥200 mL) and receipted blood transfusions or used blood products within the previous 3 months; (16) had a history of surgery within 3 months before administration of the drug or had not recovered from surgery or had an anticipated surgery planned during the trial; (17) persons directly associated with this trial; (18) poor compliance or other issues that the investigator deemed unsuitable for the trial. According to the ICF, subjects have the right to withdraw at any stage of the trial without any penalty or impact on their future medical services.

### 4.3. Study Design

This was a randomized, double-blind, placebo-controlled single ascending dose (SAD) study to evaluate the safety, tolerability, and pharmacokinetics of (R)-ketamine hydrochloride injection in Chinese healthy subjects after a single intravenous administration, including a tolerability test and a pharmacokinetic test.

This SAD study consisted of five dose cohorts (i.e., 10.0, 30.0, 60.0, 120, and 180 mg), and ten subjects (including two treated with placebo) were included in each dose cohort. The trial was conducted sequentially from the lower dose cohorts to the higher dose cohorts, and the trial of a higher dose cohort could proceed only after assessment and confirmation that the current dose was safely tolerated. Moreover, all subjects needed to fast overnight for 10 h before dosing and received a single dose intravenous administration of (R)-ketamine hydrochloride injection or placebo over 40 min (±1 min) on Day 1. No water was allowed within 2 h before and after the infusion, and no food was permitted within 4 h after the completion of the infusion.

In this study, the study drug and placebo were packaged and labeled identically in appearance to ensure that both investigators and subjects were blinded. In other words, the subjects, investigators, the sponsor, and all medical personnel involved in the treatment or clinical assessment were unaware of which investigational drug was being administered in this study (except the independent statistical team). On emergency occasions, investigators could unblind the subjects urgently after communicating with the sponsor if they thought it would be helpful to manage adverse events. In addition, according to the discontinuation criteria for dose escalation, subjects with adverse events were unblinded if any of the following criteria occurred: (1) at least two thirds of the subjects in any dose cohort experienced moderate or above severity of adverse events possibly related to the investigational drug; (2) at least two subjects in any dose cohort occurred severe adverse events possibly related to the investigational drug; (3) at least one subject in any dose cohort experienced serious adverse events possibly related to the investigational drug. Each dose cohort was randomized separately. In each dose cohort, ten subjects were randomized in a 4:1 ratio such that eight subjects received (R)-ketamine hydrochloride injection and two subjects received the placebo. The randomization list was generated by using the SAS statistical package (version 9.4, SAS Institute Inc., Cary, NC, USA).

This study was conducted in compliance with the principles of the Declaration of Helsinki and the International Conference on Harmonization Good Clinical Practice guidelines. The study protocol and ICF were approved by the Beijing Anding Hospital Capital Medical University Ethics Committee (Beijing, China). The trial was registered at http://www.chinadrugtrials.org.cn with the identifier CTR20232056, accessed on 7 July 2023.

### 4.4. Sample Collection

Blood samples were collected from all dose cohorts for pharmacokinetic assessment of (R)-ketamine and its metabolite (R)-norketamine. The venous blood samples were taken at 0 h (within −-1 h) before dosing, immediately after the completion of infusion (the acceptable delay should not exceed 30 s), and 5, 10, 15, 20, 30, 40, 50 min (±1 min), 1, 2, 3, 4 h (±3 min), 6, 8, 12 h (±5 min), 24, 36, 48, 72 h (±10 min) after the completion of infusion. Approximately 4 mL of venous blood was collected in a 4 mL vacuum blood collection vessel containing K_2_EDTA anticoagulant. After blood collection, it needs to be centrifuged within 60 min to obtain plasma. Conditions of centrifugation were 4 °C, 2000× *g* and 10 min. The obtained plasma was aliquoted into two cryogenic storage tubes. The first tube contained at least 1.0 mL of plasma for analytical testing, and the remaining plasma was allocated into the backup tube. All the plasma samples were stored at −80 °C until analysis.

### 4.5. Pharmacokinetic Assessments

The assay of (R)-ketamine and its metabolite (R)-norketamine in human plasma was performed by ultra-performance liquid chromatography tandem mass spectrometry method (UPLC-MS/MS) using multiple reaction monitoring (MRM) detection and electrospray ionization in the positive mode (ESI^+^). The ion reactions for quantitative analysis were *m*/*z* 238.1→*m*/*z* 124.9 [(R)-ketamine], *m*/*z* 224.0→*m*/*z* 124.9 [(R)-norketamine], *m*/*z* 241.1→*m*/*z* 207.1 [deuterated (R)-ketamine, internal standard]. UPLC was performed with ACQUITYTM Ultra Performance LC (Waters, Milford, MA, USA). Mass spectrometric detection was performed with Q-TRAP 6500 (AB Sciex, Foster, CA, USA). The method was fully validated. The validated calibration ranges were 0.500~500 ng/mL for both (R)-ketamine and (R)-norketamine. The data acquisition and processing were performed by Analyst software (version 1.6.3, AB Sciex, Foster, CA, USA) and Watson LIMS software (version 7.4.2, Thermo Fisher Scientific, Waltham, MA, USA), respectively.

The PK parameters of (R)-ketamine and (R)-norketamine were calculated by Phoenix^TM^ WinNonlin software (version 8.2, Certara L.P., Princeton, NJ, USA) using the non-compartmental model analysis (NCA) method. Safety statistics and PK analysis were performed using the SAS statistical package (version 9.4, SAS Institute Inc., Cary, NC, USA). The PK parameters of (R)-ketamine and (R)-norketamine included maximum drug concentration in plasma (C_max_), the time to reach C_max_ (T_max_), area under the concentration–time curve from zero to the last measurable concentration (AUC_0–t_) and infinity (AUC_0–∞_), clearance rate (CL), apparent volume of distribution (V_d_), elimination rate constant (λ_z_), elimination half-life (t_1/2_), mean residence time from time zero to the time of the last measurable concentration (MRT_0–t_) and infinity (MRT_0–∞_), area under the moment curve from time zero to the time of the last measurable concentration (AUMC_0–t_) and infinity (AUMC_0–∞_), the ratio of C_max_ of the metabolite to the parent drug (MPratio_C_max_) and the ratio of AUC_0–∞_ of the metabolite to the parent drug (MPratio_AUC_0–∞_). The relationship between the dose and PK parameters of exposure (AUC_0–t_, AUC_0–∞_ and C_max_) to (R)-ketamine and (R)-norketamine was assessed using the method combining the log-linearized power model and the confidence interval (CI) criterion to preliminarily determine whether it is consistent with linear pharmacokinetic characteristics [26]. The model can be described as ln(PK) = β_0_ + β_1_ × ln(Dose), PK = e^β0^ × Dose^β1^, where β_0_ was intercept and β_1_ was slope. When the (1-α) % CI of the β_1_ falls completely within the judgment interval, the PK parameters of exposure can be linearly related to the administered dose. In this trial, the significance level α = 0.05. Therefore, dose proportionality was confirmed when the 95% CI of the β_1_ value included the value of 1.000.

### 4.6. Safety and Tolerability Assessments

During this trial, all the subjects were required to be hospitalized for 5 days. All subjects were observed for safety and tolerability for 4 days after drug administration. If the obtained PK characteristics showed that the t_1/2_ of (R)-ketamine hydrochloride injection was significantly different from that expected, the observation duration of subjects in the subsequent dose cohorts should be adjusted to evaluate the safety and tolerability of (R)-ketamine hydrochloride injection at each dose level. A trial of a higher dose cohort could proceed only after assessment and confirmation that the current dose was safely tolerated.

By using the standard system MedDRA version 26.0 (Chinese), safety and tolerability were assessed by measurements of adverse events (AEs) including adverse drug reaction (ADR), serious adverse events (SAEs) and treatment-emergent adverse events (TEAEs), changes in clinical safety-related parameters: physical examination, vital signs (body temperature, respiration, blood pressure and pulse rate), clinical laboratory examination (blood routine, blood biochemistry, urine routine, coagulation function, blood pregnancy or urine pregnancy examination), 12-lead electrocardiogram, modified Observer’s Vigilance/Sedation Assessment Scale (MOAA/s), Clinician Rating Separation State Scale (CADSS), etc. According to the specific data values defined for the severity of adverse events in the Clinical Data Interchange Standards Consortium (CDISC) standards, the severity of AEs were graded as follows: (1) mild: signs and symptoms can be perceived by the subjects but are easily tolerated and have no significant effect on activities of daily living (ADL); (2) moderate: subjects had discomfort that affected ADL or required therapeutic intervention; (3) severe: significantly effect on the ADL of subjects, and urgent intervention indicated.

According to GuangDong Pharmaceutical Association, China: Guangdong Consensus on Safety Evaluation of Drug Clinical Trials (2020 Edition), the relationship between adverse events and the investigational drug was categorized as related, probably related, possibly related, possibly unrelated, and unrelated [27]. For the tolerability assessment, stopping criteria for dose escalation were as follows: (1) at least two thirds of the subjects in any dose cohorts experienced moderate AE possibly related to the investigational drug; (2) at least two subjects in any dose cohorts experienced severe AE possibly related to the investigational drug; (3) at least one subject in any dose cohorts experienced SAE possibly related to the investigational drug.

## 5. Conclusions

The pharmacokinetics, safety, and tolerability of (R)-ketamine hydrochloride injection were evaluated in healthy Chinese subjects following single intravenous administration. The clinical study results indicated that it is a good candidate for the treatment of TRD and laid a foundation for further clinical studies of (R)-ketamine hydrochloride in patients.

## Data Availability

The original contributions presented in this study are included in the article. Further inquiries can be directed to the corresponding authors.
